# Supplementary material for: Arboreal twig-nesting ants form dominance hierarchies over nesting resources
Source: PeerJ. 2019 Nov 27;7:e8124. doi: 10.7717/peerj.8124 (PMC6884992; doi:10.7717/peerj.8124)
Supplement: Supplemental Information 4 — R scripts to calculate dominance rankings, interaction networks, tables and histograms. [file peerj-07-8124-s004.pdf]

```
# ---
# title: "Dominance ranking"
# author: "Senay Yitbarek"
# date: "7/25/2019"
# output:
#   pdf_document: default
#   html_document: default
# ---

# Script follows practical guide by Sánchez-Tójar, A., J. Schroeder, and D. R.
Farine. 2018. A practical guide for inferring reliable dominance hierarchies and
estimating their uncertainty. Journal of Animal Ecology 87:594–608.

# clear memory
rm(list=ls())
# packages needed for this analysis
library(aniDom)
library(compete)

#Setup correct working directory.

# retrieve dataset
#ants = read.csv("/Users/senayyitbarek/Desktop/script/twigants.csv",header=TRUE,
row.names=1)

# visualize the top right 3 by 3 part of the matrix
ants[c(1:3),c(1:3)]

# Test whether the row names match the column names. Whether the same individuals
are represented.
table(names(ants)==row.names(ants))

# number of individuals that interacted
table(rowSums(ants)+colSums(ants)!=0)

# number of interactions recorded
sum(ants)

# ratio of interactions to individuals
round(sum(ants)/(table(rowSums(ants)+colSums(ants)!=0)),1)


# simulating the values. a and b are randomly set to 30 and 5,
respectively.Notice that the choice does not affect the results as we are only
interestedin exploring how the proportion of known dyads changes with the ratio
of interactions to individuals.
# This process can take a few minutes depending on the size of the database.
avalues <- c(30) #the value does not affect the result in this case
bvalues <- c(10) #the value does not affect the result in this case
N.inds.values <- c(table(rowSums(ants)+colSums(ants)!=0))
N.obs.values <- c(sum(ants)/(table(rowSums(ants)+colSums(ants)!=0)))
poiss <- c(TRUE)
dombias <- c(FALSE)
# creating empty database
db.sim <- data.frame(Ninds=integer(),
```

```
                    Nits=integer(),
                    poiss=logical(),
                    dombias=logical(),
                    unknowndyads=numeric(),
                    stringsAsFactors=FALSE)


for (simnum in 1:500){
  output <- generate_interactions(N.inds.values,
                                  N.inds.values*N.obs.values,
                                  a=avalues,
                                  b=bvalues,
                                  id.biased=poiss,
                                  rank.biased=dombias)

  # generating sociomatrix and estimating number of
  matrix<-get_wl_matrix(output$interactions)
  unknowndyads<-rshps(matrix)$unknowns/rshps(matrix)$total

  # adding values to db
  db.sim<-rbind(db.sim,c(N.inds.values,N.obs.values,
                        poiss,dombias,
                        unknowndyads))
}

names(db.sim) <- c("Ninds","N.obs.values",
                   "poiss","dombias",
                   "unknowndyads")
db.sim$knowndyads <- 1-db.sim$unknowndyad

# mean proportion of known dyads expected under the Poisson process
round(mean(db.sim$knowndyads),2)

# 2.5 and 97.5 quantiles proportion of known dyads expected under the
Poissonprocess
round(quantile(db.sim$knowndyads,probs=c(0.025,0.975)),2)

# observed proportion of known dyads
1-round(sparseness(ants),2)

# First, transforming matrix into random sequence of interactions
ants.1 <- ants
dom.data <- data.frame(interact.number=1:sum(ants.1),
                       winner=NA,loser=NA)
ids <- rownames(ants.1)
count <- 1
for (i in 1:nrow(ants.1)) {
  for (j in 1:ncol(ants.1)) {
    while (ants.1[i,j] > 0) {
      dom.data$winner[count] <- ids[i]
      dom.data$loser[count] <- ids[j]
      ants.1[i,j] <- ants.1[i,j]-1
      count <- count + 1
    }
  }
}
```

```
# hierarchy based on randomized Elo-rating
scores <- elo_scores(winners=dom.data$winner,
                     losers=dom.data$loser,
                     identities = row.names(ants),
                     randomise = TRUE,
                     n.rands = 1000,
                     return.as.ranks = TRUE)
rank <- rowMeans(scores)
rank <- rank[order(rank)]
rank

ids <- names(rank)
ranks <- 1:length(rank)
op <- par(mar = c(4,4,3,0.5))
shape <- plot_hierarchy_shape(fitted=TRUE,
                              ids,ranks,
                              dom.data$winner,
                              dom.data$loser)
#text(4,0.53,"Study:\nYitbarek & Philpott 2017",adj = 0,cex=0.75)

# Uncertainty/steepness based on Elo-rating repeatability
rept <- estimate_uncertainty_by_repeatability(dom.data$winner,
                                              dom.data$loser,
                                              identities=ids,
                                              init.score=0,
                                              n.rands = 1000)
round(rept,3)


# Uncertainty/steepness based on half comparison
halve <- estimate_uncertainty_by_splitting(dom.data$winner,
                                           dom.data$loser,
                                           identities=ids,
                                           init.score=0,
                                           randomise=TRUE,
                                           n.rands = 1000)
round(halve,2)

# Triangle transitivity
round(ttri_test(ants)$ttri,2)

# and its p-value
ttri_test(ants)$pval


# Plot network of interactions
library(igraph)
#ants =
read.csv("/Users/senayyitbarek/Desktop/Ecosphere/script/twigants.csv",header=TRUE,
 row.names=1, check.names=FALSE)
m=as.matrix(ants)
net=graph.adjacency(m,mode="directed",weighted=TRUE,diag=FALSE)
plot.igraph(net,vertex.size= 30,
vertex.label=V(net)$name,layout=layout.fruchterman.reingold,
vertex.label.color="black",edge.color="black",edge.width=E(net)$weight/3,
```

```
edge.arrow.size=1)


# Table of species rankings
library(knitr)
options(knitr.table.format = "html")
library(kableExtra)
ants=read.csv(file='table.csv', sep=',',header=T)
ants %>%
  kable() %>%
  kable_styling(bootstrap_options = "striped", full_width = F)


# Plot histogram
library(ggplot2)
df=ants
head(df)
p=ggplot(df, aes(x=reps)) + geom_histogram(color='black', fill="white")
p # plot histogram

# add mean
p+ geom_vline(aes(xintercept=mean(reps, na.rm=T)),
              +               color="red", linetype="dashed", size=1)
p+ geom_vline(aes(xintercept=mean(reps, na.rm=T)),color="red", linetype="dashed",
size=1)
```
