# Supplementary material for: Arboreal twig-nesting ants form dominance hierarchies over nesting resources
Source: PeerJ. 2019 Nov 27;7:e8124. doi: 10.7717/peerj.8124 (PMC6884992; doi:10.7717/peerj.8124)
Supplement: Supplemental Information 5 [file peerj-07-8124-s005.pdf]

Supplementary Material

Methods

Between 2008-2013, we surveyed twig-nesting ants in 6-8 hectares of a 45-ha plot and in 0-2 hectares of a 7-ha plot within Finca Irlanda in Chiapas, Mexico. In each hectare, we surveyed ants in two types of plots. First, we sampled ants in full hectare plots (100 x 100 m) and sampled ants on the coffee plant nearest to each tagged tree within that plot (alternating by survey year to N, S, E, then W of the tree). Second, we sampled ants in 20 x 20 m plots randomly placed within each hectare, and then searched for ants on every coffee plant within those plots (alternating the location of the plot within the hectare each year). For all surveys, we sampled ants by removing all dry twigs from coffee plants, counting hollow and occupied twigs, and identifying all occupant ants. The numbers of plots surveyed each year varied due to logistical constraints, but considering each plot sampled in each year as a replicate, we conducted 56 full hectare plot surveys and 44 20 x 20 m plot surveys, for a total of 100 plots surveyed. After destructive sampling of twigs, all ants and nest pieces were left at the sample location to facilitate recolonization of new nest sites by the ants.

With the ant field survey data, we calculated relative abundance of each ant species at the plot- and farm-scale. To calculate plot-scale relative abundance for each ant species, we calculated the proportion of occupied coffee plants that were occupied by that species in each full hectare or 20 x 20 m plot. Then, we averaged these values for only those plots where that ant species occurred. To calculate farm-scale relative abundance for each ant species, we calculated the proportion of occupied coffee plants that were occupied by that species in each full hectare or 20 x 20 m plot.

Then, we averaged these values across all plots sampled in the 45 ha and 7 ha sites. Because most ant species encountered are polydomous, we considered relative abundance at the scale of individual coffee plants (and not individual twigs), as a surrogate for individual ant colonies.

We next examined whether deviation in plot- vs. farm-scale relative abundance could be explained by differences in dominance rank of each ant species. First, we ran a simple linear regression between plot- and farm-scale relative abundance values, and extracted the residuals from that regression. Then, we correlated the regression residuals with the Elo-rating (dominance rank) values for each species to examine both if (1) dominance ranking significantly explained higher plot-scale abundance (compared to abundance at the farm-scale) and (2) what fraction of the variation in plot- vs. farm-scale abundance was explained by dominance ranking.

Results

We found that deviations from farm-scale abundance at the plot- scale were significantly correlated to dominance ranking ($F=6.27$, $df=1, 8$, $P=0.037$, Figure S1). That is, those species with low Elo-rating (stronger competitors) were more abundant at the plot-scale compared with their abundance at the farm-scale, and those ant species with high Elo-rating (weaker competitors) were less abundant at the plot-scale compared with their relative abundance at the farm scale. However, although significant, the dominance ranking only explained 43.94% of the variation in the relationship, indicating that dominance rank, although likely important, is only one of many factors contributing to relative abundance patterns of these ants.

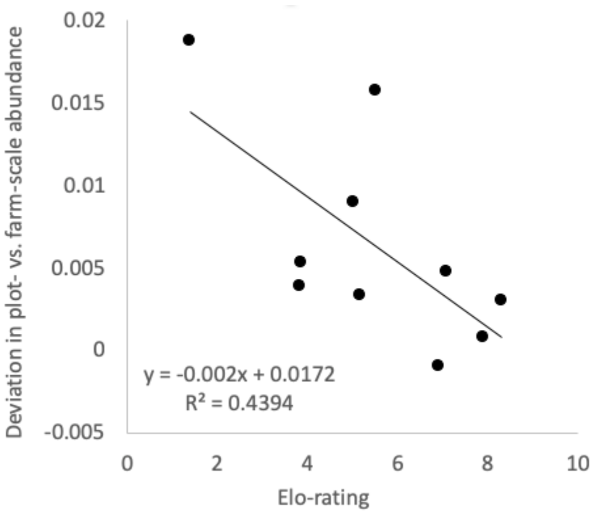

Figure S1. Relationship between Elo-rating (dominance rank) and deviation in plot- vs. farm-scale abundance patterns for a community of arboreal twig-nesting ants in coffee agroecosystems. Each point represents a single ant species where y-axis values above 0 show higher relative abundance at the local plot-scale compared with relative abundance across all plots sampled (farm-scale).
